# Metabolic and Risk Profiles of Lean and Non-Lean Hepatic Steatosis among US Adults

**DOI:** 10.3390/nu15132856

**Published:** 2023-06-23

**Authors:** Meiling Li, Weiping Zhang, Xiude Li, Shaoxian Liang, Yaozong Zhang, Yufeng Mo, Songxian Rao, Honghua Zhang, Yong Huang, Yu Zhu, Zhuang Zhang, Wanshui Yang

**Affiliations:** 1 Department of Nutrition, School of Public Health, Anhui Medical University, 81 Meishan Road, Hefei 230032, China; 2045010416@stu.ahmu.edu.cn (M.L.); 2045010421@stu.ahmu.edu.cn (W.Z.); 2046010029@stu.ahmu.edu.cn (X.L.); 2145010436@stu.ahmu.edu.cn (S.L.); 2145010450@stu.ahmu.edu.cn (Y.Z.); 2145010439@stu.ahmu.edu.cn (Y.M.); 2145010441@stu.ahmu.edu.cn (S.R.); 2145010321@stu.ahmu.edu.cn (H.Z.); 2146010036@stu.ahmu.edu.cn (Y.H.); 2046010043@stu.ahmu.edu.cn (Y.Z.); 2020500011@ahmu.edu.cn (Z.Z.); 2 Key Laboratory of Population Health Across Life Cycle, Anhui Medical University, Ministry of Education of the People’s Republic of China, Hefei 230032, China; 3 NHC Key Laboratory of Study on Abnormal Gametes and Reproductive Tract, Hefei 230032, China; 4 Anhui Provincial Key Laboratory of Population Health and Aristogenics/Key Laboratory of Environmental Toxicology of Anhui Higher Education Institutes, Anhui Medical University, Hefei 230032, China

**Keywords:** steatosis, lean, etiological heterogeneity

## Abstract

Hepatic steatosis can occur in lean individuals, while its metabolic and risk profiles remain unclear. We aimed to characterize the clinical and risk profiles of lean and non-lean steatosis. This cross-sectional study included 1610 patients with transient elastography-assessed steatosis. The metabolic and risk profiles were compared. Compared to their non-lean counterparts, lean subjects with steatosis had a lower degree of fibrosis (F0–F1: 91.9% vs. 80.9%), had a lower prevalence of diabetes (27.9% vs. 32.8%), dyslipidemia (54.7% vs. 60.2%) and hypertension (50.0% vs. 51.3%), and had higher levels of high-density lipoprotein cholesterol while lower fasting insulin and homeostatic model assessment for insulin resistance (all *p* < 0.05). Of the 16 potential risk factors, being Hispanic was associated with higher odds of non-lean steatosis but not with lean steatosis (odds ratio (OR): 2.07 vs. 0.93), while excessive alcohol consumption had a different trend in the ratio (OR: 1.47 vs.6.65). Higher waist-to-hip ratio (OR: 7.48 vs. 2.45), and higher waist circumference (OR: 1.14 vs. 1.07) showed a stronger positive association with lean steatosis than with non-lean steatosis (all *P*_heterogeneity_ < 0.05). Although lean individuals with steatosis presented a healthier metabolic profile, both lean and non-lean steatosis had a significant proportion of metabolic derangements. In addition, the etiological heterogeneity between lean and non-lean steatosis may exist.

## 1. Introduction

Non-alcoholic fatty liver disease (NAFLD) is the leading cause of chronic liver disease, with a prevalence of approximately 32.4% globally. Hepatic steatosis is an essential early histopathological feature of NAFLD. Roughly 25–30% of US adults have hepatic steatosis. NAFLD is generally considered as the hepatic manifestation of the metabolic syndrome and is particularly common in subjects with type 2 diabetes and obesity. However, increasing evidence showed that such disease exists in lean individuals, namely, lean NAFLD (i.e., NAFLD patients with a body mass index (BMI) below the ethnic-specific cut-offs of 23 kg/m^2^ in Asians and 25 kg/m^2^ in non-Asians) [1], which constitutes over 40% of the NAFLD population [2]. In addition, several [2,3,4] but not all studies [5,6] suggested both lean and non-lean NAFLD may have had substantial long-term hepatic and extrahepatic comorbidities [2,3] and a similar risk of progressing cardiovascular diseases (CVDs) and malignancies [4]. Moreover, few studies showed that lean NAFLD patients had a higher long-term risk for the development of severe liver disease [7] and mortality [8] compared to patients with NAFLD and a higher BMI. Despite being debatable, these findings underscore that lean individuals with NAFLD should not be overlooked in clinical practice.

Nonetheless, the clinical and metabolic profiles of NAFLD, particularly lean NAFLD, are poorly understood. Several studies showed that lean NAFLD patients had a healthier metabolic profile and lower disease severity compared to non-lean counterparts, which somewhat contradicts the findings of some studies that lean patients had a similar or even worse prognosis [4,7,8], while other studies [9,10] showed that they did not. The inconsistency could have been partly due to the difference in study populations. Previous studies were predominantly conducted on Asians [11,12], while data on Americans is scarce [13]. Asians are likely to have more central fat deposition and thereby tend to develop NAFLD and other metabolic disorders at a lower BMI. Moreover, NAFLD patients in most studies were recruited from healthcare clinics and determined by liver biopsy [5,14], which were highly selected and may, therefore, hamper the representativeness and lead to selection bias, although the liver biopsy remains the gold standard for diagnosis of NAFLD.

Beyond the metabolic profiles of NAFLD, another concern is whether lean and non-lean NAFLD have different risk profiles. Dietary modification (e.g., shift to a Mediterranean diet) and increased physical activity to achieve weight loss are highly recommended for the prevention and management of NAFLD. However, there have been no specific guidelines for lean NAFLD because of the lack of evidence on its risk profiles, despite few data [15] indicating that there are differences in factors affecting lean and non-lean NAFLD, such as demographic information, body measurement data, diet, metabolic factors, and other lifestyle factors.

Herein, we aimed to characterize the clinical and metabolic profiles of lean and non-lean hepatic steatosis, including the prevalence, metabolic biomarkers and disorders, liver function and inflammation biomarkers, stage of steatosis, and degree of fibrosis in a national representative sample from the National Health and Nutrition Examination Survey (NHANES) 2017–2018. We noted that two early studies using NHANES III [13] or NHANES 1999–2016 data [16] have reported the prevalence and mortality outcome of lean or non-obese NAFLD, in which NAFLD was diagnosed based on ultrasound and the US fatty liver index, respectively. Different from such approaches, we used vibration-controlled transient elastography (VCTE), a noninvasive method to define steatosis with higher sensitivity and specificity [17]. To investigate whether there are any differences in risk profiles, we also compared the associations of potential risk factors with lean and non-lean steatosis.

## 2. Materials and Methods

### 2.1. Study Population

**Figure 1:**
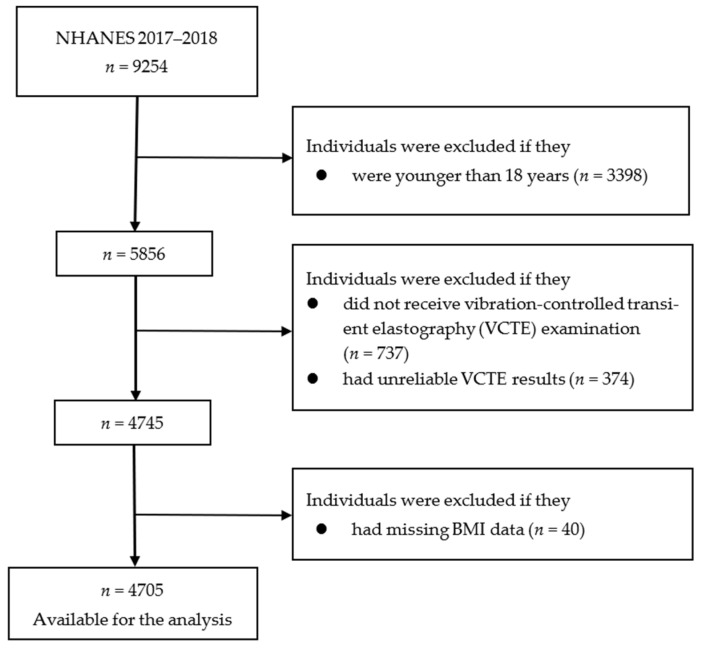
Flowchart of the selection of participants in the analysis. BMI, body mass index; NHANES, National Health and Nutrition Examination Survey; VCTE, vibration-controlled transient elastography.

NHANES is a nationwide cross-sectional survey that combines in-person interviews with standardized physical examinations and laboratory tests. Details of NHANES study design, study protocol, and methodology of data collection are available elsewhere [18]. Written informed consent was obtained from all participants. The US National Center for Health Statistics Research Ethics Review Board approved the NHANES study protocols (Protocol #2011-17; Protocol #2018-01). We excluded 3398 individuals who were younger than 18 years. Participants were additionally excluded if they had no (*n* = 737) or unreliable (*n* = 374) VCTE examination data or had missing BMI data (*n* = 40). Thus, 4705 participants were included in the final analysis (Figure 1).

### 2.2. Assessments of Lifestyle and Other Factors

We identified several possible risk factors, including age, sex, race, education, income, physical activity, Alternate Mediterranean Diet Index (AMED), smoking, drinking, coffee intake, waist-to-hip ratio (WHR), waist circumference (WC), energy, diabetes, hypertension, and dyslipidemia for lean and non-lean steatosis by reviewing the literature (Supplementary Table S1). Age, sex, race/ethnicity, education, smoking, physical activity, and income were ascertained by household interviews with standardized questionnaires. Information on alcohol drinking, height, body weight, WC, and hip circumference was obtained during the NHANES mobile examination center visit. Dietary information was collected using single or two 24 h dietary recall(s). A total of 44.4% of participants (*n* = 2090) completed the two dietary recalls, with the first recall being conducted via face-to-face interview at the NHANES Mobile Examination Center and the second recall being conducted via phone call 3–10 days after the first one. To improve the completeness and accuracy of the food recall and reduce the respondent burden, multiple-pass method approaches were used.

Family income was measured by the ratio of family income to poverty. Excessive alcohol consumption was defined as ≥3 standard drinks per day on average for men and ≥2 for women. BMI was calculated as weight in kilograms divided by the square of the height in meters (kg/m^2^). WHR was calculated as WC divided by hip circumference, and high WHR was defined as ≥0.9 for men and ≥0.85 for women. Meeting the physical activity guideline was defined as achieving the World Health Organization (WHO) recommended physical activity levels (i.e., ≥150 min/week of moderate-intensity physical activity, ≥75 min/week of vigorous-intensity physical activity, or an equivalent combination).

### 2.3. Assessments of Plasma Biomarkers

Hepatitis B virus (HBV) infection was indicated by a positive surface antigen test and hepatitis C virus (HCV) infection was defined as both hepatitis C antibody and ribonucleic acid positive. Hypertension was identified through a self-reported diagnosis of hypertension, a systolic blood pressure (SBP) level ≥ 140 mmHg, or a diastolic blood pressure (DBP) level ≥ 90 mmHg. Diabetes was defined through a self-reported diagnosis of diabetes, a fasting glucose level ≥ 126 mg/dL, or a hemoglobin A1c (HbA1c) level ≥ 6.5%. Dyslipidemia was defined as total lipoprotein cholesterol (TC) ≥ 200 mg/dL, low-density lipoprotein cholesterol (LDL-C) ≥ 130 mg/dL, triglyceride (TG) ≥ 150 mg/dL, non-high-density lipoprotein cholesterol (non-HDL-C) ≥ 160 mg/dL, or HDL-C ≤ 40 mg/dL. Homeostatic model assessment for insulin resistance (HOMA-IR) was obtained using the following formula: HOMA-IR = fasting insulin (uU/L) × fasting glucose (mmol/L)/22.5. Laboratory methods of measuring these biochemical indicators were described elsewhere [18].

### 2.4. Definition of Liver Diseases

In the 2017–2018 cycle of NHANES, the VCTE using the FibroScan^®^ model 502 V2 Touch (Echosens, Paris, France) test equipped with a medium (M) or extra-large (XL) wand (probe) was performed by trained technicians. Consistent with the prior study [17], we used controlled attenuation parameter (CAP) cut-off values of 274, 290, and 302 (dB/m) to define S1, S2, and S3 steatosis, respectively. We also used liver stiffness measurement (LSM) cut-off values of no less than 8.2, 9.7, and 13.6 (kPa) to define F2 (significant fibrosis), F3 (advanced fibrosis), and F4 (liver cirrhosis), respectively. A reliable VCTE examination was considered only when more than 10 LSMs were obtained after a fasting time of ≤3 h, with an interquartile range to median ratio of <30%. Lean steatosis individuals were identified as individuals with steatosis and a BMI of ≤23 kg/m^2^ for Asians and ≤25 kg/m^2^ for non-Asians [1].

### 2.5. Statistical Analysis

We used appropriate sampling weights, stratification, and clustering of the complex sampling design for each analysis [16]. The steatosis prevalence was standardized by age using the 2000–2025 WHO standard population (single ages until 79 and then 80 years or older). The method that compared metabolic and clinical features between lean and non-lean steatosis was as follows: significance was tested using the Student’s *t*-test for continuous parameters if normally distributed, and with the Kruskal-Wallis test if non-normally distributed.The multivariable logistic regression model was used to calculate the odds ratios (ORs) and 95% confidence intervals (CIs). The heterogeneity between lean and non-lean steatosis associated with the potential risk factors was detected using Cochran’s Q test. 

Models were adjusted for sex (male, female), age (18–39, 40–59, or ≥60 years), race/ethnicity (Hispanic, non-Hispanic, or other races), total energy intake (kcal/day, tertile), education (<12th grade, high school graduate, or more than high school), the ratio of family income to poverty (<1.30, 1.30–3.49, or ≥3.50), meeting the physical activity guideline (no, yes), smoking (never smoking, former smoking, or current smoking), alcohol drinking (never drinking, former drinking, or current drinking), diabetes (no, yes), HBV infection (no, yes), HCV infection (no, yes), hypertension (no, yes), dyslipidemia (no, yes), BMI (continuous, kg/m^2^), WC (continuous, cm), high WHR (no, yes), AMED (score, tertile), and coffee intake (g/day, tertile). Since alcohol drinking was a component of AMED score, alcohol drinking was removed from the AMED score when alcohol drinking and AMED score simultaneously entered the model. We did not adjust for alcohol drinking when investigating the AMED variable. A missing-value indicator was created for covariates with missing values in the models. All analyses were conducted using SAS version 9.4 (SAS Institute Inc., Cary, NC, USA) and R software (version 4.1.3), and a two-tailed value of *p* < 0.05 was considered statistically significant.

## 3. Results

### 3.1. Characteristics of Participants

Of the 4705 participants (mean (standard deviation SD) age, 49.3 (18.3) years), 1610 (age-standardized prevalence, 31.7%) were diagnosed with hepatic steatosis (CAP ≥ 290 dB/m). The prevalence of steatosis was 7.1% among lean subjects (below the ethnic-specific cut-offs of 23 kg/m^2^ in Asians and 25 kg/m^2^ in non-Asians) and 45.0% among non-lean subjects. The prevalence of lean steatosis was 7.9% in Caucasians, 7.8% in Hispanics, 9.1% in Asians, and 3.9% in Blacks. Compared to non-lean steatosis, lean individuals with steatosis were older (age: 57.8 vs. 52.6 years), had higher AMED scores (3.9 vs. 3.3), had a lower prevalence of high WHR (81.4% vs. 92.7%), lower WC (88.2 vs. 112.1 cm), a lower degree of fibrosis (F0–F1: 91.9% vs. 80.9%), a lower prevalence of diabetes (27.9% vs. 32.8%), dyslipidemia (54.7% vs. 60.2%), and hypertension (50.0% vs. 51.3%) (Table 1).

### 3.2. Metabolic and Clinical Profile of Lean and Non-Lean Steatosis

Lean individuals with hepatic steatosis had lower levels of HOMA-IR, fasting insulin, and high levels of HDL-C compared to those with non-lean steatosis (all *p* < 0.05, Table 2).

### 3.3. Risk Profile of Lean and Non-Lean Steatosis

Being female and following the physical activity guideline were inversely associated with the odds of hepatic steatosis, whereas Hispanic ethnicity, excessive alcohol consumption, high WHR, higher WC, diabetes, hypertension, and dyslipidemia were positively associated with the odds of hepatic steatosis (Table 3).

In the heterogeneity analysis, Hispanic ethnicity was associated with a higher risk of non-lean steatosis (OR: 2.07, 95% CI: 1.59–2.69) but not with lean steatosis (OR: 0.93, 95% CI: 0.62–1.38, *P*_heterogeneity_ = 0.001). Excessive alcohol consumption showed a strong association with the likelihood of lean steatosis (OR: 6.65, 95% CI: 2.07–21.37) but a moderate association with non-lean steatosis (OR: 1.47, 95% CI: 0.98–2.22, *P*_heterogeneity_ = 0.017). Higher WHR presented a stronger positive association with lean steatosis (OR: 7.48, 95% CI: 3.52–15.92) than with non-lean steatosis (OR: 2.45, 95% CI:1.60–3.73, *P*_heterogeneity_ = 0.011). Similarly, the positive association of WC with lean steatosis (OR:1.14, 95% CI: 1.09–1.20) was stronger than that with non-lean steatosis (OR: 1.07, 95% CI:1.05–1.10, *P*_heterogeneity_ = 0.020).

## 4. Discussion

In this study, we compared the metabolic and risk profiles between lean and non-lean steatosis in a nationally representative sample of US adults. The prevalence of steatosis in lean individuals was 7.1% and varied widely by ethnicity/race, being 7.9% in Caucasians, 7.8% in Hispanics, 9.1% in Asians, and 3.9% in blacks. Compared to individuals with non-lean steatosis, lean individuals with steatosis generally had healthier metabolic profiles. As for the risk profile, associations for Hispanic ethnicity, excessive alcohol consumption, high WHR, and high WC showed evident heterogeneity between lean and non-lean steatosis. Given a significant proportion of the long-term severe hepatic and extrahepatic outcomes in patients with lean steatosis, our findings reveal the high prevalence of lean steatosis in a nationwide community-dwelling population and provide clues for a specific guideline for lean steatosis prevention.

We found that the prevalence of steatosis in lean individuals was 7.1% in a US nationwide community-based population. Despite limited reports regarding the prevalence of lean steatosis in free-living populations, our results were similar to those in a few existing population-based studies showing that among lean subjects the prevalence of NAFLD determined by ultrasound was 7.4% in the NHANES III study and the prevalence of NAFLD determined by the fatty liver index was 9.6% in the 1999–2016 NHANES study [13,16]. Moreover, a cohort study including subjects from Italy, UK, Spain, and Australia suggested that the prevalence of biopsy-proven NAFLD was 14.4% in Caucasians with a BMI of <25 kg/m^2^ [3]. In China, of 731 subjects with a BMI of <24 kg/m^2^, 18.3% had ultrasonographic evidence of NAFLD [9], whereas in another study of 29,994 Korean health check, nonobese participants, 12.6% had NAFLD [12].

We found that the prevalence of lean steatosis differed across race/ethnicity, with higher prevalence among Asians and Caucasians and lowest among Blacks, which may be attributed to the differences in genetic susceptibility and body fat distribution. First, the most essential gene involved in the development of hepatic steatosis is the patatin-like phospholipase domain-containing 3 (PNPLA3). An allele in PNPLA3-(rs738409[G] encoding L148M) was related to an elevated risk of hepatic steatosis, and the prevalence of PNPLA3 rs738409 ranged by race/ethnicity [19]. Second, given that hepatic steatosis was closely correlated with visceral adipose tissue (VAT), racial/ethnic differences in VAT may interpret the variation in steatosis. For example, a study demonstrated that ethnic differences in liver fat between large samples of African-American, Hispanic, and Caucasian adults were entirely removed after adjusting for the differences in visceral fat but not after adjusting for total fat and abdominal subcutaneous adipose tissue [20]. These factors are not mutually exclusive and may happen and act jointly. 

We found that lean steatosis had substantial proportions of metabolic derangements, albeit with a “healthier” metabolic profile than that with non-lean steatosis. Lean subjects with steatosis had lower HOMA-IR compared to the non-lean counterparts with steatosis in the present study. The evidence showed that obesity is a risk factor for the development of insulin resistance. Fasting insulin and insulin resistance are closely related, which may partly explain why the lean subjects in this study had lower levels of fasting insulin. However, lean/non-obese NAFLD patients may have a similar risk of developing metabolic disease as obese NAFLD. Lean NAFLD patients had a markedly higher prevalence of high risk for atherosclerotic cardiovascular disease than obese NAFLD [21]. Therefore, lean individuals with steatosis cannot be discounted and more research is needed to study lean steatosis. In addition, we also found HDL-C was higher in lean individuals than in non-lean individuals with steatosis. TG was lower in lean individuals than in non-lean individuals with steatosis, although the difference was not significant. Notably, High TG and low HDL-C were intermediate markers for fatty liver [22]. In line with the previous study of lean NAFLD [23], in the current study, lean individuals with steatosis were older, had a lower degree of fibrosis, and had a lower prevalence of diabetes, hyperlipidemia, and hypertension. These results illustrated that lean steatosis has a more favorable clinical profile than non-lean steatosis.

Our results showed that being Hispanic was associated with higher odds of steatosis and that this association was only found in non-lean steatosis and not in lean steatosis, which is based in part on the higher prevalence of obesity and insulin resistance in this ethnic group [24]. In the present study, non-lean steatosis had higher levels of insulin resistance than lean subjects, which was coherent with other studies of lean NAFLD [23,25]. Taken together, this partially accounts for the stronger positive association of Hispanic individuals with non-lean steatosis. Confirmation is necessary for other racial/ethnic groups and regions.

Excessive alcohol consumption is a risk factor for hepatic steatosis, which is caused in part by the generation of excess reducing equivalents from ethanol metabolism, which enhances the accumulation of fat. Our analysis showed that the positive association between excessive alcohol consumption and steatosis was stronger among lean individuals. One of the possible reasons is that diabetes is one of the risk factors for steatosis. The relationship between alcohol consumption and type 2 diabetes also varied by BMI. For example, one research observed an inverse relationship between alcohol consumption and diabetes in overweight or obese subjects [26]. However, another study showed a positive correlation between alcohol consumption and diabetes in lean persons [27]. Therefore, attention should be paid to the effects of excessive alcohol consumption when developing guidelines for the prevention and treatment of lean subjects with steatosis.

We found that both WHR and WC showed stronger associations with lean steatosis than that with non-lean steatosis. Previous research found a saturation effect of waist-to-height ratio (WHtR) on NAFLD, with a significant increase in the risk of NAFLD after a WHtR of approximately 0.4 and no or only a small increase in the risk of NAFLD after a WHtR of roughly 0.6 [28]. WHR, WC, and WHtR reflect central obesity to some extent. Therefore, WHR and WC may also have saturation effects on steatosis. However, it cannot be excluded that it was found by chance. The specific mechanism remains to be further confirmed in future studies.

The strengths of our study include the use of a large nationally representative sample of US adults and a reliable VCTE examination with a high sensitivity and specificity for measuring hepatic steatosis [17]. However, we also realized several limitations. First, data from the questionnaire was self-reported and may introduce measurement errors. Second, due to limited cases of lean steatosis in the analysis, chance findings cannot be excluded. Third, the cross-sectional design in the current study is unable to determine the causation. 

## 5. Conclusions

In summary, although lean individuals with steatosis generally showed a healthier metabolic profile, both lean and non-lean steatosis had a significant proportion of metabolic derangements. Heavy alcohol drinking and increased WC or WHR showed a more prominent positive association with the likelihood of lean steatosis than that with non-lean steatosis. Prospective cohort studies are needed to validate these findings.

## Figures and Tables

**Table 1:** Characteristics of the study population in NHANES (2017–2018) ^†^.

Characteristic	Total (*n* = 4705)	Lean Steatosis (*n* = 86)	Non-Lean Steatosis (*n* = 1524)	*p* *
No. of steatosis, %	1610 (34.2)	-	-	
Age, years	49.3 (18.3)	57.8 (17.0)	52.6 (16.5)	0.004
Female, %	50.5	43.0	42.9	0.975
BMI, kg/m^2^	29.4 (7.1)	23.0 (1.6)	34.2 (6.8)	<0.001
Race/ethnicity, %				0.062
Hispanic	23.2	18.6	29.4	
Non-Hispanic white	33.9	41.9	35.6	
Non-Hispanic black	22.9	12.8	17.4	
Non-Hispanic Asian	14.5	19.8	12.7	
Other races	5.5	7.0	4.9	
Education, %				0.847
≤12th grade	19.5	18.6	20.8	
High school graduate/GED or equivalent	24.9	26.7	25.8	
More than high school	55.4	54.7	53.1	
Ratio of family income to poverty, %				0.643
<1.3	25.3	30.2	24.9	
1.3 to 3.5	35.2	32.6	36.0	
≥3.5	26.8	23.3	26.6	
Meeting the physical activity guideline, %	63.1	55.8	59.3	0.725
AMED score	3.4 (1.8)	3.9 (2.1)	3.3 (1.7)	0.009
Smoking, %				0.642
Never smoking	59.9	57.0	55.6	
Former smoking	22.8	30.2	27.9	
Current smoking	17.3	12.8	16.5	
Drinking, %				0.032
Never drinking	10.7	15.1	9.8	
Former drinking	19.0	23.3	21.2	
Current drinking	65.1	52.3	64.7	
Coffee intake, %	58.5	55.8	55.8	0.097
High WHR, %	75.5	81.4	92.7	0.002
WC, cm	99.4 (16.7)	88.2 (7.4)	112.1 (14.3)	<0.001
Total energy, kcal/d	2037 (880)	1990 (998)	2073 (847)	0.470
History of diseases, %				
Diabetes	18.9	27.9	32.8	0.345
Hypertension	41.3	50.0	51.3	0.512
Dyslipidemia	48.7	54.7	60.2	0.295
HBV	0.6	0.0	0.9	0.533
HCV	1.8	2.3	1.6	0.749
CAP				
Median ^‡^	262	311	328	<0.001
S0	2677 (56.9)	-	-	
S1	418 (8.9)	-	-	
S2	301 (6.4)	32 (37.2)	269 (17.7)	<0.001
S3	1309 (27.8)	54 (62.8)	1255 (82.3)	
LSM				
Median ^‡^	4.9	5.1	5.8	0.001
F0–F1	4257 (90.5)	79 (91.9)	1233 (80.9)	0.007
F2	163 (3.5)	6 (7.0)	102 (6.7)	
F3	166 (3.5)	0 (0.0)	115 (7.6)	
F4	119 (2.5)	1 (1.2)	74 (4.9)	

AMED, Alternate Mediterranean Diet Index; BMI, body mass index; CAP, controlled attenuation parameter; GED, general educational development; HBV, hepatitis B virus; HCV, hepatitis C virus; LSM, liver stiffness measurement; NHANES, National Health and Nutrition Examination Survey; SD, standard deviation; WC, waist circumference; WHR, waist-to-hip ratio. ^†^ Continuous variables were expressed as mean (SD) if normally distributed and as median if non-normally distributed. Categorical variables were expressed as proportions (%). Values of polytomous variables may not sum to 100% due to missing values or rounding. ^‡^ The numbers in brackets represent percentages. * Test between lean and non-lean steatosis. Significance was tested with the Student’s *t*-test for continuous parameters if normally distributed and with the Kruskal–Wallis test if non-normally distributed. Significance was tested with the Chi-square test for categorical parameters and with the Kruskal–Wallis test for ranked data.

**Table 2:** Metabolic biomarkers between lean and non-lean steatosis in NHANES (2017–2018) ^†^.

Metabolic Biomarkers	Lean Steatosis (*n* = 86)	Non-Lean Steatosis (*n* = 1524)	*p*
TC	176 (158, 198)	178 (164, 192)	0.815
TG	85 (61, 118)	100 (82, 122)	0.144
HDL-C	54 (48, 60)	47 (42, 51)	0.003
LDL-C	74 (56, 99)	81 (65, 101)	0.233
DBP	64 (56, 72)	66 (61, 71)	0.422
SBP	124 (116, 132)	126 (119, 132)	0.549
ALT	19.12 (15.27, 23.94)	22.45 (19.02, 26.50)	0.071
AST	22.95 (18.89, 27.88)	21.24 (18.31, 24.63)	0.267
GGT	32.77 (22.30, 48.17)	34.99 (26.62, 45.98)	0.591
ALP	83.51 (69.44, 100.45)	78.61 (66.19, 93.37)	0.236
hs-CRP	0.17 (0.10, 0.28)	0.21 (0.14, 0.33)	0.288
HOME–IR	2.72 (1.67, 4.44)	4.57 (3.10, 6.82)	0.008
Fasting blood glucose	6.76 (5.45, 8.38)	6.23 (5.35, 7.24)	0.317
Fasting insulin	9.16 (6.40, 13.10)	16.73 (12.42, 22.56)	0.001
Hemoglobin A1c	6.30 (5.98, 6.62)	6.19 (6.02, 6.37)	0.434

ALP, alkaline phosphatase; ALT, alanine aminotransferase; AST, aspartate aminotransferase; DBP, diastolic blood pressure; GGT, gamma-glutamyl transferase; HDL-C, high-density lipoprotein cholesterol; HOME-IR, homeostatic model assessment for insulin resistance; hs-CRP, high-sensitivity C-reactive protein; LDL-C, low-density lipoprotein cholesterol; SBP, systolic blood pressure; TC, total cholesterol; TG, triglycerides. ^†^ Significance was tested with the Student’s *t*-test for continuous parameters if normally distributed and with the Kruskal–Wallis test if non-normally distributed.

**Table 3:** Heterogeneity between lean steatosis and non-lean steatosis in NHANES (2017–2018) ^†^.

Variable (Reference)	OR (95% CI)			*P*_heterogenity_ ^‡^
	Total Steatosis	Lean Steatosis	Non-Lean Steatosis	
Age (18–59 years)	1.29 (0.88–1.89)	1.43 (0.71–2.88)	1.27 (0.86–1.89)	0.772
Sex (male)	0.49 (0.39–0.61)	0.44 (0.22–0.92)	0.48 (0.38–0.61)	0.821
Race (other races)	1.90 (1.46–2.48)	0.93 (0.62–1.38)	2.07 (1.59–2.69)	0.001
Education (high school or below)	0.92 (0.73–1.18)	1.33 (0.55–3.18)	0.92 (0.70–1.19)	0.431
Ratio of family income to poverty (<2.5)	1.10 (0.83–1.45)	0.64 (0.31–1.30)	1.17 (0.87–1.57)	0.127
Meeting the physical activity guideline (no)	0.75 (0.59–0.96)	1.06 (0.54–2.06)	0.73 (0.56–0.95)	0.310
AMED score (below the mean score)	0.92 (0.69–1.24)	0.98 (0.50–1.93)	0.93 (0.70–1.24)	0.889
Smoking (never)	1.12 (0.88–1.42)	1.08 (0.47–2.46)	1.10 (0.86–1.41)	0.967
Excessive alcohol consumption (no)	1.95 (1.14–3.33)	6.65 (2.07–21.37)	1.47 (0.98–2.22)	0.017
Coffee intake (no)	0.97 (0.79–1.19)	0.73 (0.38–1.42)	1.02 (0.82–1.26)	0.344
High WHR (no)	2.48 (1.66–3.71)	7.48 (3.52–15.92)	2.45 (1.60–3.73)	0.011
WC (continuous)	1.07 (1.05–1.09)	1.14 (1.09–1.20)	1.07 (1.05–1.10)	0.020
Energy (below the mean level)	1.10 (0.93–1.31)	0.66 (0.39–1.12)	1.15 (0.95–1.38)	0.052
Diabetes (no)	2.58 (1.86–3.58)	3.51 (1.43–8.64)	2.37 (1.73–3.25)	0.419
Hypertension (no)	1.44 (1.10–1.89)	1.37 (0.63–2.98)	1.46 (1.16–1.83)	0.878
Dyslipidemia (no)	1.58 (1.28–1.95)	1.27 (0.63–2.57)	1.64 (1.30–2.08)	0.499

AMED, Alternate Mediterranean Diet Index; BMI, body mass index; CI, confidence intervals; GED, general educational development; HBV, hepatitis B virus; HCV, hepatitis C virus; NHANES, National Health and Nutrition Examination Survey; OR, odds ratio; WC, waist circumference; WHR, waist-to-hip ratio. ^†^ Models were adjusted for sex (male, female), age (18–39, 40–59, or ≥60 years), race/ethnicity (Hispanic, non-Hispanic, or other races), total energy intake (kcal/day, tertile), education (<12th grade, high school graduate/GED or equivalent, or more than high school), ratio of family income to poverty (<1.30, 1.30–3.49, or ≥3.50), meeting the physical activity guideline (no, yes), smoking (never smoking, former smoking, or current smoking), alcohol drinking (never drinking, former drinking, or current drinking), diabetes (no, yes), HBV infection (no, yes), HCV infection (no, yes), hypertension (no, yes), dyslipidemia (no, yes), BMI (continuous, kg/m^2^), WC (continuous, cm), high WHR (no, yes), AMED (score, tertile), and coffee intake (g/day, tertile). ^‡^ Heterogeneity between lean and non-lean steatosis. Due to the limited cases, the variables examined in etiological heterogeneity were transformed into binary variables in the regression model, except for WC, which was treated as a continuous variable. These variables included sex (male, female), age (18–59, ≥60 years), race/ethnicity (Hispanic, other races), total energy intake (below the mean level, above or equal to the mean level), education (high school or below, more than high school), ratio of family income to poverty (<2.5, ≥2.50), meeting the physical activity guideline (no, yes), smoking (never, ever), excessive alcohol consumption (no, yes), diabetes (no, yes), HBV infection (no, yes), HCV infection (no, yes), hypertension (no, yes), dyslipidemia (no, yes), high WHR (no, yes), AMED (below the mean score, above or equal to the mean score), and coffee intake (no, yes). To avoid over-adjustment, alcohol drinking was removed from the AMED score when testing variables other than AMED. When testing the AMED variable, alcohol drinking was removed from the models.

## Data Availability

Data described in the manuscript, code book, and analytic code will be made publicly and freely available without restriction at https://www.cdc.gov/nchs/nhanes/index.htm (accessed on 6 June 2023).

## Supplementary Materials

The following supporting information can be downloaded at https://www.mdpi.com/article/10.3390/nu15132856/s1. Supplementary Table S1; Selection of influencing factors for chronic liver disease. Refs. [29,30,31,32,33,34,35,36,37,38,39,40,41,42,43,44,45,46,47,48,49,50,51,52,53,54,55,56,57] have been cited in Supplementary Materials.
