# Supplementary material for: Metabolic and Risk Profiles of Lean and Non-Lean Hepatic Steatosis among US Adults
Source: Nutrients. 2023 Jun 23;15(13):2856. doi: 10.3390/nu15132856 (PMC10343664; doi:10.3390/nu15132856)
Supplement: Supplementary file 1 [file nutrients-15-02856-s001.zip › nutrients-2465758-supplementary.pdf]

Supplementary Table S1. Selection of influencing factors for chronic liver disease

Influence factors	Authors (year)	Country	Design	Outcome	Measurement	HR/OR/RR (95% CI)	References
Age	Hamaguchi et al. (2012)	Japan	Cohort study	NAFLD	Abdominal ultrasonography	OR: 1.03 (1.00–1.06)	[29]
	Shaheen et al. (2021)	USA	Cohort study	Hepatic steatosis	FibroScan	AOR: 1.81 (1.27–2.57)	[30]
Sex	Summart et al. (2017)	Thailand	Cross-sectional study	NAFLD	Ultrasonography	OR: 1.30 (1.20–1.40)	[31]
	Shaheen et al. (2021)	USA	Cohort study	Hepatic steatosis	FibroScan	AOR: 0.41 (0.31–0.54)	[30]
Race	Shaheen et al. (2021)	USA	Cross-sectional study	NAFLD	FibroScan	AOR: 2.40 (1.40–4.20)	[32]
	Rehm et al. (2014)	USA	Cross-sectional study	Hepatic steatosis	Magnetic resonance imaging proton density fat fraction	OR: 4.26 (1.65–11.04)	[33]
Education	Vilar-Gomez et al. (2021)	USA	Cross-sectional study	NAFLD	FibroScan	OR: 0.65 (0.43–0.96)	[34]
	Fan et al. (2021)	China	Cross-sectional study	MAFLD	Ultrasonography	OR: 1.50 (1.23–1.82)	[35]
Income	Fan et al. (2021)	China	Cross-sectional study	MAFLD	Ultrasonography	OR: 1.22 (1.05–1.42)	[35]
	Huo et al. (2021)	China	Cohort study	NAFLD	Ultrasonography	HR: 1.72 (1.11–2.66)	[36]
Physical activity	Gerage et al. (2018)	Brazil	Cohort study	Hepatic steatosis	Ultrasonography	OR: 0.71 (0.57–0.88)	[37]
	Fan et al. (2021)	China	Cross-sectional study	MAFLD	Ultrasonography	OR: 0.71 (0.60–0.85)	[35]
AMED scores	Khalatbari-Soltani et al. (2019)	Finland	Cohort study	Hepatic steatosis	Abdominal ultrasonography	PR: 0.67 (0.56–0.78)	[38]
	Doustmohammadian et al. (2022)	Iran	Cross-sectional study	NAFLD	Abdominal sonography	OR: 0.64 (0.52–0.78)	[39]
Smoking	Charatcharoenwitthaya et al. (2020)	Thailand	Cohort study	NAFLD	Ultrasonography	HR: 2.09 (1.18–3.71)	[40]
	Wu et al. (2021)	Finland	Cohort study	NAFLD	Ultrasonography	RR: 1.99 (1.14–3.45)	[41]

Drinking	Long et al. (2020)	USA	Cross-sectional study	Hepatic steatosis	Multidetector computed tomography	OR: 1.15 (1.02–1.29)	[42]
	Lau et al. (2015)	USA	Cross-sectional study	Hepatic steatosis	Ultrasonography	OR: 1.36 (1.06–1.74)	[43]
Coffee	Imatoh et al. (2015)	Japan	Cross-sectional study	Hepatic steatosis	Ultrasonography	OR: 0.59 (0.38–0.90)	[44]
	Veronese et al. (2018)	South Italy	Cross-sectional study	Hepatic steatosis	Ultrasonography	OR: 0.93 (0.72–1.20)	[45]
Waist-to-hip ratio	Schult et al. (2018)	Sweden	Cohort study	Liver cirrhosis	Hospital discharge and death certificate registries	HR: 5.80 (1.60–21.40)	[46]
	Andreasson et al. (2017)	Sweden	Cohort study	Liver disease	Swedish national registers	HR: 1.30 (1.16–1.46)	[47]
Waist circumference	Campbell et al. (2016)	USA	Cohort study	Liver cancer	State cancer registries, medical record abstraction, and/or the National Death Index	HR: 1.08 (1.04–1.13)	[48]
	Ju et al. (2013)	South Korea	Cross-sectional study	NAFLD	Abdominal ultrasonography	Men<br>OR: 15.54 (12.69–19.03)<br>Women<br>OR: 48.73 (23.92–99.29)	[49]
Energy	Wehmeyer et al. (2016)	Germany	Cohort study	NAFLD	Ultrasonography	–	[50]
	Anderson et al. (2015)	England	Cohort study	NAFLD	Ultrasonography	OR: 0.88 (0.66–1.17)	[51]
Diabetes	Ming et al. (2015)	China	Cohort study	NAFLD	Abdominal ultrasonography	RR: 4.50 (1.90–10.70)	[52]
	Kleiner et al. (2019)	USA	Cohort study	Severe steatosis	Liver biopsies	RR: 2.04 (1.10–3.76)	[53]
Hypertension	Gawrieh et al. (2019)	USA	Cross-sectional study	NASH	Biopsy specimens	OR: 1.50 (0.90–2.50)	[54]
	Aneni et al. 2015	USA	Cross-sectional study	NAFLD	Abdominal ultrasonography	OR: 1.80 (1.40–2.30)	[55]
Dyslipidemia	Kathak et al. (2022)	Bangladesh	Cross-sectional study	Liver disease	Liver enzymes	–	[56]
	Mendez-Sanchez et al. (2020)	Mexico	Cross-sectional study	Liver fibrosis	Biopsy-proven	OR: 0.81 (0.71–1.61)	[57]

AMED, Alternate Mediterranean Diet Index; AOR, adjusted odds ratio; CI, Confidence interval; HR, Hazard ratio; MAFLD, metabolic dysfunction-associated fatty liver disease; NAFLD, Nonalcoholic fatty liver disease; NASH, Non-alcoholic steatohepatitis; OR, Odds ratio; PR, Prevalence ratio; RR, Risk ratio.

References

29. Hamaguchi, M.; Kojima, T.; Ohbora, A.; Takeda, N.; Fukui, M.; Kato, T. Aging is a risk factor of nonalcoholic fatty liver disease in premenopausal women. *World J Gastroenterol* **2012**, *18*, 237-243, doi:10.3748/wjg.v18.i3.237.
30. Shaheen, M.; Pan, D.; Schrode, K.M.; Kermah, D.; Puri, V.; Zarrinpar, A.; Elisha, D.; Najjar, S.M.; Friedman, T.C. Reassessment of the Hispanic Disparity: Hepatic Steatosis Is More Prevalent in Mexican Americans Than Other Hispanics. *Hepatol Commun* **2021**, *5*, 2068-2079, doi:10.1002/hep4.1775.
31. Summart, U.; Thinkhamrop, B.; Chamadol, N.; Khuntikeo, N.; Songthamwat, M.; Kim, C.S. Gender differences in the prevalence of nonalcoholic fatty liver disease in the Northeast of Thailand: A population-based cross-sectional study. *F1000Res* **2017**, *6*, 1630, doi:10.12688/f1000research.12417.2.
32. Shaheen, M.; Schrode, K.M.; Pan, D.; Kermah, D.; Puri, V.; Zarrinpar, A.; Elisha, D.; Najjar, S.M.; Friedman, T.C. Sex-Specific Differences in the Association Between Race/Ethnicity and NAFLD Among US Population. *Front Med (Lausanne)* **2021**, *8*, 795421, doi:10.3389/fmed.2021.795421.
33. Rehm, J.L.; Connor, E.L.; Wolfgram, P.M.; Eickhoff, J.C.; Reeder, S.B.; Allen, D.B. Predicting hepatic steatosis in a racially and ethnically diverse cohort of adolescent girls. *J Pediatr* **2014**, *165*, 319-325 e311, doi:10.1016/j.jpeds.2014.04.019.
34. Vilar-Gomez, E.; Nephew, L.D.; Vuppalanchi, R.; Gawrieh, S.; Mladenovic, A.; Pike, F.; Samala, N.; Chalasani, N. High-quality diet, physical activity, and college education are associated with low risk of NAFLD among the US population. *Hepatology* **2022**, *75*, 1491-1506, doi:10.1002/hep.32207.
35. Fan, J.; Luo, S.; Ye, Y.; Ju, J.; Zhang, Z.; Liu, L.; Yang, J.; Xia, M. Prevalence and risk factors of metabolic associated fatty liver disease in the contemporary South China population. *Nutr Metab (Lond)* **2021**, *18*, 82, doi:10.1186/s12986-021-00611-x.
36. Huo, Y.B.; Bai, Y.N.; Zhang, D.S.; Chang, X.Y.; Yin, C.; Ba, Y.P.; Wang, Y.F.; Gan, T.; Ding, J.; Li, N., et al. [Analysis on influencing factors for nonalcoholic fatty liver disease in Jinchang cohort]. *Zhonghua Liu Xing Bing Xue Za Zhi* **2021**, *42*, 493-498, doi:10.3760/cma.j.cn112338-20200428-00668.
37. Gerage, A.M.; Ritti-Dias, R.M.; Balagopal, P.B.; Conceicao, R.D.O.; Umpierre, D.; Santos, R.D.; Cucato, G.G.; Bittencourt, M.S. Physical activity levels and hepatic steatosis: A longitudinal follow-up study in adults. *J Gastroenterol Hepatol* **2018**, *33*, 741-746, doi:10.1111/jgh.13965.
38. Khalatbari-Soltani, S.; Imamura, F.; Brage, S.; De Lucia Rolfe, E.; Griffin, S.J.; Wareham, N.J.; Marques-Vidal, P.; Forouhi, N.G. The association between adherence to the Mediterranean diet and hepatic steatosis: cross-sectional analysis of two independent studies, the UK Fenland Study and the Swiss CoLaus Study. *BMC Med* **2019**, *17*, 19, doi:10.1186/s12916-019-1251-7.
39. Doustmohammadian, A.; Clark, C.C.T.; Maadi, M.; Motamed, N.; Sobhrakhshankhah, E.; Ajdarkosh, H.; Mansourian, M.R.; Esfandyari, S.; Hanjani, N.A.; Nikkhoo, M., et al. Favorable association between Mediterranean diet (MeD) and DASH with NAFLD among Iranian adults of the Amol Cohort Study (AmolCS). *Sci Rep* **2022**, *12*, 2131, doi:10.1038/s41598-022-06035-8.
40. Charatcharoenwitthaya, P.; Karaketklang, K.; Aekplakorn, W. Cigarette Smoking Increased Risk of Overall Mortality in Patients With Non-alcoholic Fatty Liver Disease: A Nationwide Population-Based Cohort Study. *Front Med (Lausanne)* **2020**, *7*, 604919, doi:10.3389/fmed.2020.604919.
41. Wu, F.; Pahkala, K.; Juonala, M.; Jaakkola, J.; Rovio, S.P.; Lehtimaki, T.; Sabin, M.A.; Jula, A.; Hutri-Kahonen, N.; Laitinen, T., et al. Childhood and Adulthood Passive Smoking and Nonalcoholic Fatty Liver in Midlife: A 31-year Cohort Study. *Am J Gastroenterol* **2021**, *116*, 1256-1263, doi:10.14309/ajg.0000000000001141.
42. Long, M.T.; Massaro, J.M.; Hoffmann, U.; Benjamin, E.J.; Naimi, T.S. Alcohol Use Is Associated With Hepatic Steatosis Among Persons With Presumed Nonalcoholic Fatty Liver Disease. *Clin Gastroenterol Hepatol* **2020**, *18*, 1831-1841 e1835, doi:10.1016/j.cgh.2019.11.022.
43. Lau, K.; Baumeister, S.E.; Lieb, W.; Meffert, P.J.; Lerch, M.M.; Mayerle, J.; Volzke, H. The combined effects of alcohol consumption and body mass index on hepatic steatosis in a general population sample of European men and women. *Aliment Pharmacol Ther* **2015**, *41*, 467-476, doi:10.1111/apt.13067.
44. Imatoh, T.; Kamimura, S.; Miyazaki, M. Coffee but not green tea consumption is associated with prevalence and severity of hepatic steatosis: the impact on leptin level. *Eur J Clin Nutr* **2015**, *69*, 1023-1027, doi:10.1038/ejcn.2015.23.
45. Veronese, N.; Notarnicola, M.; Cisternino, A.M.; Reddavide, R.; Inguaggiato, R.; Guerra, V.; Rotolo, O.; Zinzi, I.; Leandro, G.; Correale, M., et al. Coffee Intake and Liver Steatosis: A Population Study in a Mediterranean Area. *Nutrients* **2018**, *10*, 89, doi:10.3390/nu10010089.
46. Schult, A.; Mehlig, K.; Bjorkelund, C.; Wallerstedt, S.; Kaczynski, J. Waist-to-hip ratio but not body mass index predicts liver cirrhosis in women. *Scand J Gastroenterol* **2018**, *53*, 212-217, doi:10.1080/00365521.2017.1420219.
47. Andreasson, A.; Carlsson, A.C.; Onnerhag, K.; Hagstrom, H. Waist/Hip Ratio Better Predicts Development of Severe Liver Disease Within 20 Years Than Body Mass Index: A Population-based Cohort Study. *Clin Gastroenterol Hepatol* **2017**, *15*, 1294-1301 e1292, doi:10.1016/j.cgh.2017.02.040.

48. Campbell, P.T.; Newton, C.C.; Freedman, N.D.; Koshiol, J.; Alavanja, M.C.; Beane Freeman, L.E.; Buring, J.E.; Chan, A.T.; Chong, D.Q.; Datta, M., et al. Body Mass Index, Waist Circumference, Diabetes, and Risk of Liver Cancer for U.S. Adults. *Cancer Res* **2016**, *76*, 6076-6083, doi:10.1158/0008-5472.CAN-16-0787.
49. Ju, D.Y.; Choe, Y.G.; Cho, Y.K.; Shin, D.S.; Yoo, S.H.; Yim, S.H.; Lee, J.Y.; Park, J.H.; Kim, H.J.; Park, D.I., et al. The influence of waist circumference on insulin resistance and nonalcoholic fatty liver disease in apparently healthy Korean adults. *Clin Mol Hepatol* **2013**, *19*, 140-147, doi:10.3350/cmh.2013.19.2.140.
50. Wehmeyer, M.H.; Zyriax, B.C.; Jagemann, B.; Roth, E.; Windler, E.; Schulze Zur Wiesch, J.; Lohse, A.W.; Kluwe, J. Nonalcoholic fatty liver disease is associated with excessive calorie intake rather than a distinctive dietary pattern. *Medicine (Baltimore)* **2016**, *95*, e3887, doi:10.1097/MD.0000000000003887.
51. Anderson, E.L.; Howe, L.D.; Fraser, A.; Macdonald-Wallis, C.; Callaway, M.P.; Sattar, N.; Day, C.; Tilling, K.; Lawlor, D.A. Childhood energy intake is associated with nonalcoholic fatty liver disease in adolescents. *J Nutr* **2015**, *145*, 983-989, doi:10.3945/jn.114.208397.
52. Ming, J.; Xu, S.; Gao, B.; Liu, G.; Ji, Y.; Yang, F.; Jia, Y.; Fang, Y.; Ji, Q. Non-alcoholic fatty liver disease predicts type 2 diabetes mellitus, but not prediabetes, in Xi'an, China: a five-year cohort study. *Liver Int* **2015**, *35*, 2401-2407, doi:10.1111/liv.12851.
53. Kleiner, D.E.; Brunt, E.M.; Wilson, L.A.; Behling, C.; Guy, C.; Contos, M.; Cummings, O.; Yeh, M.; Gill, R.; Chalasani, N., et al. Association of Histologic Disease Activity With Progression of Nonalcoholic Fatty Liver Disease. *JAMA Netw Open* **2019**, *2*, e1912565, doi:10.1001/jamanetworkopen.2019.12565.
54. Gawrieh, S.; Wilson, L.A.; Cummings, O.W.; Clark, J.M.; Loomba, R.; Hameed, B.; Abdelmalek, M.F.; Dasarathy, S.; Neuschwander-Tetri, B.A.; Kowdley, K., et al. Histologic Findings of Advanced Fibrosis and Cirrhosis in Patients With Nonalcoholic Fatty Liver Disease Who Have Normal Aminotransferase Levels. *Am J Gastroenterol* **2019**, *114*, 1626-1635, doi:10.14309/ajg.0000000000000388.
55. Aneni, E.C.; Oni, E.T.; Martin, S.S.; Blaha, M.J.; Agatston, A.S.; Feldman, T.; Veledar, E.; Conceicao, R.D.; Carvalho, J.A.; Santos, R.D., et al. Blood pressure is associated with the presence and severity of nonalcoholic fatty liver disease across the spectrum of cardiometabolic risk. *J Hypertens* **2015**, *33*, 1207-1214, doi:10.1097/HJH.0000000000000532.
56. Kathak, R.R.; Sumon, A.H.; Molla, N.H.; Hasan, M.; Miah, R.; Tuba, H.R.; Habib, A.; Ali, N. The association between elevated lipid profile and liver enzymes: a study on Bangladeshi adults. *Sci Rep* **2022**, *12*, 1711, doi:10.1038/s41598-022-05766-y.
57. Mendez-Sanchez, N.; Cerda-Reyes, E.; Higuera-de-la-Tijera, F.; Salas-Garcia, A.K.; Cabrera-Palma, S.; Cabrera-Alvarez, G.; Cortez-Hernandez, C.; Perez-Arredondo, L.A.; Puron-Gonzalez, E.; Coronado-Alejandro, E., et al. Dyslipidemia as a risk factor for liver fibrosis progression in a multicentric population with non-alcoholic steatohepatitis. *F1000Res* **2020**, *9*, 56, doi:10.12688/f1000research.21918.1.
